# NLR and BRCA mutational status in patients with high grade serous advanced ovarian cancer

**DOI:** 10.1038/s41598-021-90361-w

**Published:** 2021-05-27

**Authors:** Claudia Marchetti, Marco D’Indinosante, Carolina Bottoni, Chiara Di Ilio, Stefano Di Berardino, Barbara Costantini, Angelo Minucci, Laura Vertechy, Giovanni Scambia, Anna Fagotti

**Affiliations:** 1 Department of Woman, Child and Public Health, Fondazione Policlinico Universitario A. Gemelli IRCCS, Rome, Italy; 2 Catholic University of the Sacred Heart, Rome, Italy; 3 Molecular and Genomic Diagnostics Laboratory, Fondazione Policlinico Universitario A. Gemelli IRCCS, Rome, Italy; 4 Department of Women’s and Children’s Health, Fondazione “Policlinico Universitario A.Gemelli”-IRCCS, Largo Agostino Gemelli 8, 00168 Rome, Italy

**Keywords:** Gynaecological cancer, Ovarian cancer

## Abstract

Laboratory-markers of the systemic inflammatory-response, such as neutrophil/lymphocyte-ratio (NLR) have been studied as prognostic factors in several tumors but in OC-patients their role is still controversial and no data about the possible correlation with the BRCA-status has been ever reported. We consecutively enrolled a series of 397 newly diagnosed high-grade serous-advanced OC-patients. All patients were tested for BRCA-mutational-status and blood-parameters have been collected 48 h before staging-surgery. A significant correlation of NLR with disease distribution (p < 0.005) was found and patients with NLR < 4 underwent primary-debulking-surgery more frequently (p-value 0.001), with a lower surgical-complexity-score (p-value 0.002). Regarding survival-data, patients with NLR < 4 had a significant 7-month increase in mPFS (26 vs 19 months, p = 0.009); focusing on the BRCA-status, among both BRCA-mutated and BRCA-wild type patients, those with lower NLR had a significantly prolonged mPFS compared to patients with NLR > 4 (BRCA-mutated: 35 vs 23 months, p = 0.03; BRCA-wt: 19 vs 16 months, p = 0.05). At multivariate-analysis, independent factors of prolonged PFS were BRCA mutational status, having received complete cytoreduction and NLR < 4. Also, the strongest predictors of longer OS were BRCA-mutational status, having received complete cytoreductive surgery, NLR < 4 and age. NLR is confirmed to be a prognostic marker in OC-patients and it seems unrelated with BRCA-mutational status.

## Introduction

Ovarian cancer remains the most lethal gynecological malignancy in developed countries^1^.

Nearly 75% of OC-affected women present with advanced disease (stage III or IV) and most of these will die from their disease, with 5-year overall survival rates around 30%^2^.

Several prognostic factors have been identified to predict the outcome and guide personalized treatment of patients with advanced OC, including histological type, FIGO-stage, residual tumor after surgery, response to chemotherapy, and BRCA1/2-mutation status^3^. In addition to clinical and molecular features, recent data suggest the host-driven inflammatory response's influence on tumors' behavior and treatment outcome^4,5^. In fact, tumor growth and metastatic spread result from several interactions between tumoral and stromal factors, including blood vessels, inflammatory cells and the immune system, leading to a chronic inflammation status^6,7^.

Laboratory markers of the systemic inflammatory response, such as white blood cell count, have been studied as prognostic and predictive factors in several tumors^8,9^. High NLR, defined as the absolute neutrophils count divided by the lymphocytes count^9–11^, has shown a negative prognostic impact in the stomach, colorectal cancer and other cancers^12–14^. The mechanism underlying the association between high NLR and the worse outcome has not been clarified yet. Probably, neutrophilia can inhibit the immune system by blocking the cytolytic activity of immune cells^15,16^, and promotes tumor growth by producing vascular endothelial growth factor^17,18^. On the other hand, lymphocytopenia is frequently found in patients with advanced disease, indicating a lower immune activity against tumor antigens released by cancer cells^19–21^. Moreover, lymphocytes T seem keener to apoptosis^22,23^.

In OC, inflammation markers have shown interesting but controversial results^24^. In particular, recent trials have suggested that a high NLR is correlated with an immunosuppressive profile^25^ and with poorer overall survival and could be a predictive marker for treatment efficacy^26–28^. Rising literature has also demonstrated the correlation between NLR-ratio and more advanced ovarian cancer disease, with some series suggesting that preoperative higher NLR ratio is also associated with a greater risk of 30-day postoperative morbidity^29–31^. However, there is currently no established threshold value for the neutrophil-to-lymphocyte ratio.

Albeit OCs harboring a BRCA mutation are considered more immunoreactive and with higher mutational and neoantigen loads than BRCA wild-type tumors, no study has investigated the possible correlation between NLR values and BRCA mutational status. In light of these aspects, we investigated the relationship between BRCA status and systemic inflammatory factors in a large high-grade serous OC population.

## Results

### Patients’ characteristics

Overall, 397 patients fulfilled the inclusion criteria (primary diagnosis of high-grade serous ovarian cancer (HGSOC), had received 3 weekly carboplatin-paclitaxel as first line treatment, known BRCA mutational status, see “Materials and methods” section) and were evaluable for the biomarkers of interest.

Characteristics of evaluated patients are described in Table 1. The mean age at diagnosis was 60.2 years (range 27–89); 271 (68.3%) were BRCA wild-type (BRCA-WT), with 17 (4.3%) having a BRCA variant of uncertain significance (BRCA-VUS), and 127 (31.7%) had a BRCA 1/2 pathogenetic variants (BRCA-PVs). Among the latter, 79 (19.9%) patients presented with a BRCA1 mutation, and 47 (11.8%) presented with BRCA2. At diagnosis, the median value of NLR across the overall population was 4.02 (range 0.95–33). The association between the preoperative NLR score and clinicopathologic characteristics of EOC patients is shown in Table 1.

**Table 1:** Characteristics of HGSOC patients by NLR.

	Total	Group 1 (NLR ≤ 4)	Group 2 (NLR > 4)	P-value
	N (%)	N (%)	N (%)	
All cases	397	196 (49.4)	201 (50.6)	
Mean age at diagnosis (range, years)	60.2 (27–89)	60.8 (27–89)	59.8 (30–85)	0.837
**Type of BRCA mutation**				0.97
No mutation	271 (68.3)	135 (68.3)	136 (68.3)	
BRCA1	79 (19.9)	39 (20.1)	40 (20.1)	
BRCA2	47 (11.8)	24 (12.1)	23 (11.6)	
CA125, mean (SD), UI/mL	2632 (4265)	2085 (3152)	1973 (3190)	0.81
**FIGO stage at diagnosis**^**a**^				
III	282 (71.8)	144 (73.1)	138 (70.4)	0.31
IV	111 (28.2)	53 (26.9)	58 (29.6)	
**LPS-PIV**^**b**^				
< 8	173 (43.9)	108 (55.4)	65 (32.7)	0.0001
≥ 8	221 (56.1)	87 (44.6)	134 (67.3)	
**Primary treatment strategy**				
PDS	186 (46.9)	110 (55.6)	76 (38.2)	0.001
NACT	211 (53.1)	88 (44.4)	123 (61.8)	
**Surgical complexity score**^c,^*				
1–2	82 (44.6)	59 (54.1)	23 (30.7)	0.002
3	102 (55.4)	50 (45.9)	52 (69.3)	
**RT at primary surgery (PDS)**				
0	158 (84.9)	92 (83.6)	66 (86.8)	0.75
1–10 mm	18 (9.7)	11 (10)	7 (9.1)	
> 1 cm	10 (5.4)	7 (6.4)	3 (3.9)	

WT: wild type; VUS: variants of uncertain significance; PVs: pathogenetic variants; FIGO: International Federation of Gynecology and Obstetrics; LPS-PIV: laparoscopic predictive index value; PDS: primary debulking surgery; NACT: neoadjuvant chemotherapy; RT residual tumor; NLR: neutrophile/lymphocyte ratio.

^a^Data calculated on 393 patients due to lack of data of 4 patients.

^b^Data calculated on 394 patients due to lack of data of 3 patients.

^c^Data calculated on 395 patients due to lack of data of 2 patients.

* Calculated only in women treated with PDS.

Regarding the BRCA status, no significant differences were found regarding NLR values (p-value: 0.97). The majority of patients presented as stage III of disease (282, 71.8%), without differences related to NLR value. We found a significant correlation of NLR with disease distribution, with more patients with low tumor load in Group 1 (NLR < 4) versus Group 2 (NLR > 4) (44.6% vs 67.3%) (p < 0.0001).

Moreover, patients in group 1 (110, 55.6%) underwent PDS more frequently than patients in group 2 (76, 38.2%) (p-value 0.001), with no statistically significant difference of complete/optimal cytoreduction in the overall population. Among patients undergoing PDS, those with lower NLR also had a lower surgical complexity score (59, 54.1%) compared with those in group 2 (23, 30.7%) (p-value 0.002).

### Impact of NLR on survival

The median follow-up was 24 months (range 4–47). At the time of final analysis, 210 (52.9%) of patients have recurred in the overall population, with more recurrences among group 2 (57.8%) versus group 1 (48%), respectively (p = 0.03). Similarly, 105 (24.2%) patients were dead, 64 (32.2%) in group 2 and 41 (20.7%) in group 1 (p = 0.007).

**Figure 1:**
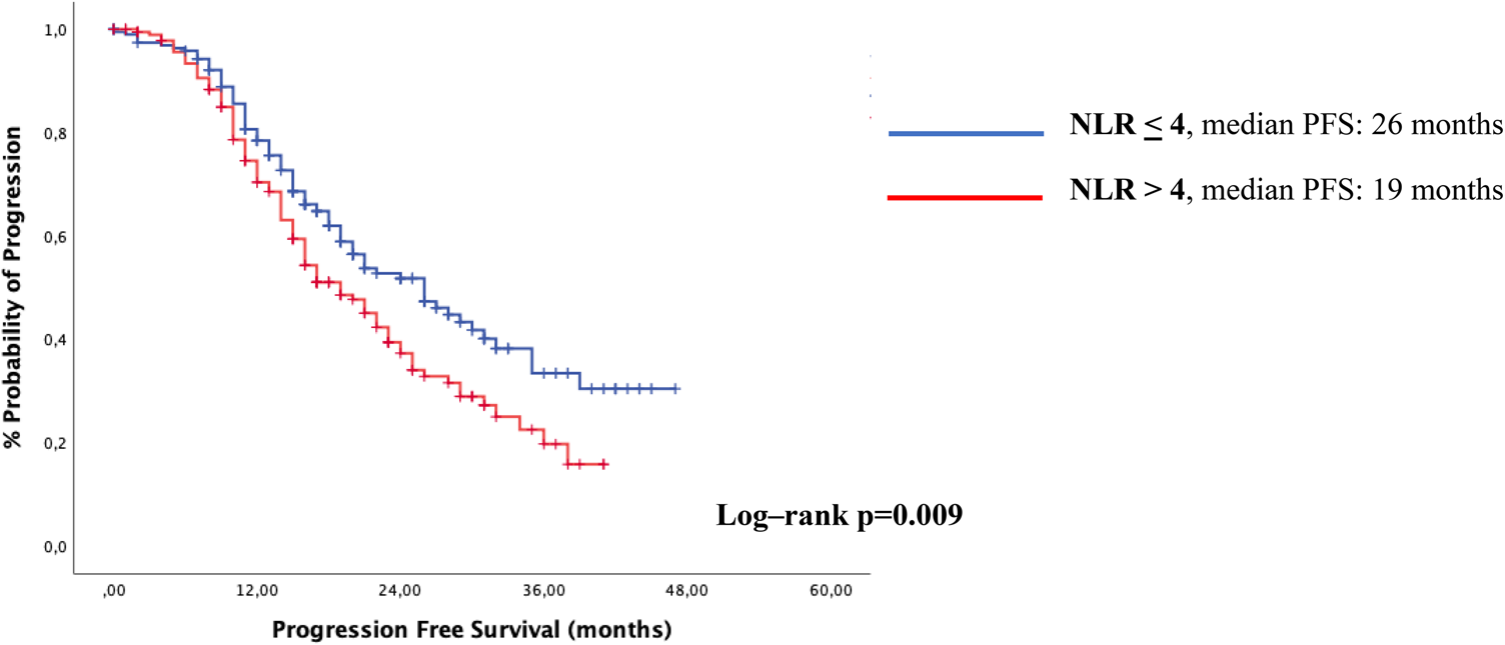
Kaplan–Meyer plots for progression free survival (PFS) according to NLR-value, overall population.

In the overall population, median progression-free survival (mPFS) was 21 months; those with NLR < 4 had a significant 7-month increase in mPFS, compared with patients with NLR > 4 (26 months vs. 19 months, p = 0.009, Fig. 1).

**Figure 2:**
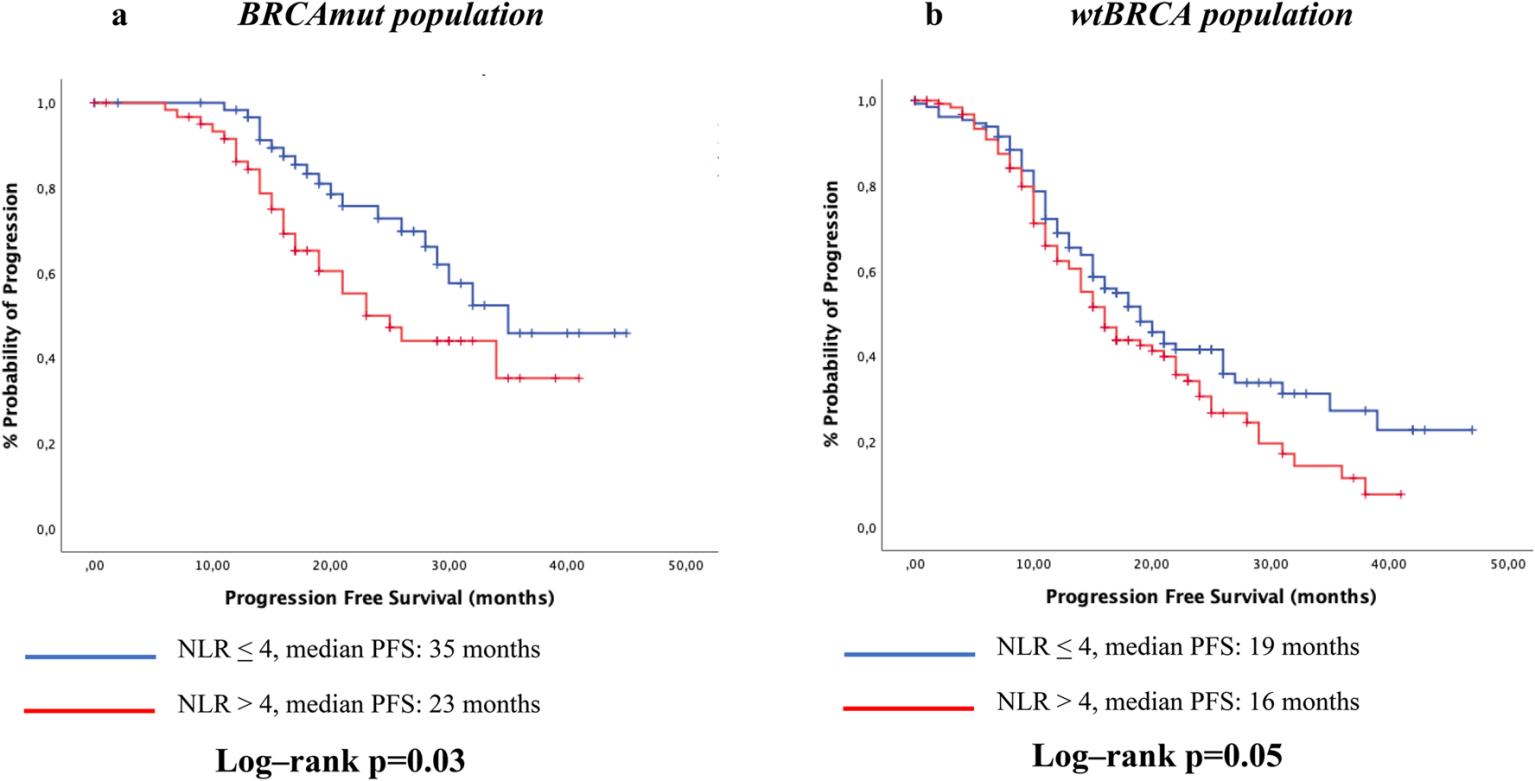
Kaplan–Meyer plots for progression free survival (PFS) according to NLR-value and BRCA status, subgroup analysis.

Focusing on the BRCA status, among BRCAmut patients, those with lower NLR had a significantly prolonged mPFS (35 months vs. 23 months, p = 0.03, Fig. 2a); similarly, among BRCA wild-type patients, those with lower NLR had a slightly significant 3-month increase in mPFS, compared with patients in group 2 (19 months vs. 16 months, p = 0.05, Fig. 2b). At multivariate analysis for PFS, independent factors of prolonged PFS were BRCA mutational status (HR 0.50, CI 95% 0.35–0.71), having received complete cytoreduction (HR 0.51, CI 95% 0.35–0.75) and NLR < 4 (HR 0.69, CI 95% 0.51–0.95) (Table 2).

**Table 2:** Cox univariate and multivariate analysis for progression-free survival (PFS).

Variables	Univariate analysis		Multivariate analysis	
	HR (95% CI)	P-value	HR (95% CI)	P-value
Age	1.01 (1.00–1.03)	0.008	1.00 (0.09–1.02)	0.34
LPS-PIV < 8/ ≥ 8	0.61 (0.46–0.81)	0.001	0.98 (0.64–1.52)	0.96
BRCA status mut/wt	0.43 (0.31–0.60)	0.0001	0.50 (0.35–0.71)	0.0001
PDS/NACT	0.61 (0.46–0.81)	0.001	0.79 (0.52–1.20)	0.27
RT 0/ > 0	0.49 (0.33–0.71)	0.0001	0.51 (0.35–0.75)	0.001
NLR ≤ 4/ > 4	0.69 (0.52–0.91)	0.01	0.69 (0.51–0.95)	0.023

LPS-PIV: laparoscopic predictive index value; mut: mutated; WT: wild type; PDS: primary debulking surgery; NACT: neoadjuvant chemotherapy; RT: residual tumor; NLR: neutrophil/lymphocyte ratio.

**Figure 3:**
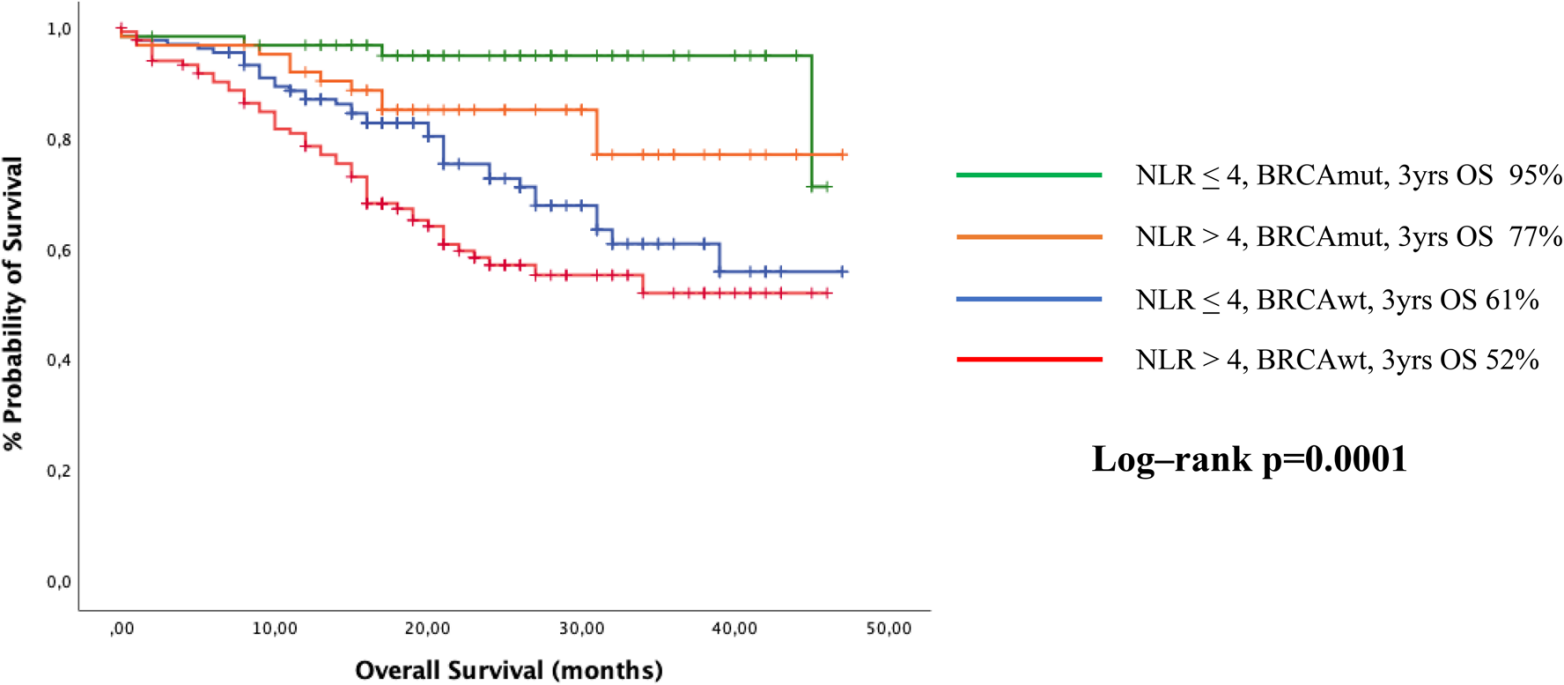
Kaplan–Meyer plots for overall survival (OS) according to NLR-value and BRCA status, overall population.

Median overall survival (mOS) at 60 months was still not reached and 3 years OS was 72% in group 1 and 60% in group 2 (p = 0.007). Statistically significant differences related to NLR were found in the BRCAmut (mOS not reached in both groups, p = 0.05), and BRCA wild-type (mOS not reached in both groups, p = 0.027) populations (Fig. 3). In the multivariate analysis, the strongest predictors of longer OS were BRCA mutational status (HR 0.47, CI 95% 0.26–0.85) having received complete cytoreduction (HR 0.42, CI 95% 0.25–0.72), NLR < 4 (HR 0.58, CI 95% 0.36–0.95) and younger age (HR 1.03, CI 95% 1.01–1.06) (Table 3).

**Table 3:** Cox univariate and multivariate analysis for overall-survival (OS).

Variables	Univariate analysis		Multivariate analysis	
	HR (95% CI)	P-value	HR (95% CI)	P-value
Age	1.05 (1.03–1.07)	0.0001	1.03 (1.01–1.06)	0.001
LPS-PIV < 8/ ≥ 8	0.46 (0.30–0.71)	0.0001	0.81 (0.39–1.65)	0.563
BRCA status mut/wt	0.29 (0.16–0.50)	0.0001	0.47 (0.26–0.85)	0.013
PDS/NACT	0.43 (0.28–0.65)	0.0001	0.79 (0.39–1.57)	0.497
RT 0/ > 0	0.40 (0.24–0.68)	0.001	0.42 (0.25–0.72)	0.001
NLR ≤ 4/ > 4	0.59 (0.39–9.87)	0.008	0.58 (0.36–0.95)	0.032

LPS-PIV: laparoscopic predictive index value; mut: mutated; WT: wild type; PDS: primary debulking surgery; NACT: neoadjuvant chemotherapy; RT: residual tumor; NLR: neutrophil/lymphocyte ratio.

## Discussion

In this study, we found for the first time that high NLR (> 4) has a negative prognostic role in patients with primary advanced OC, in terms of both PFS and OS, regardless of BRCA status.

Several evidences suggest that BRCA-mutated OC disease exhibits statistically significantly higher mutational and neoantigen loads and might be more immunologically "hot"/T-cell inflamed than BRCAwt and HR proficient ovarian cancers. Therefore, we wanted to explore whether or not a simple blood biomarker could predict this. Nevertheless, we weren't able to find such a correlation, as NLR values seem unrelated to the presence/absence of the BRCA mutation. In other words, if we assume that NLR is correlated, at least indirectly, with the immune status, based on our results, individual immunoreactivity to cancer is independent of BRCA status. We shouldn't be surprised that BRCA, as well as HRD status, do not linearly predict response to immune checkpoint inhibitors (ICIs)^32,33^ and we should consider NLR as a more reliable predictor of immunotherapy response even in OC, as it has been recently demonstrated in other cancers^34–36^.

More importantly, we came up with the evidence that OC BRCA-mutated disease is not "one disease" with peculiar and good survival outcomes. Even in the presence of a BRCA mutation, the prognosis can be determined by other factors, of which NLR is one easily identifiable. Indeed, among BRCAmut patients, those with low NLR had seven months advantage of mPFS with respect to women with high NLR.

Moreover, it is now recognized that PARPi, which are known to achieve their most remarkable efficacy in BRCA mutated and HRD cancer cells, can also get a response in HR proficient cancer^37^. This can be explained by both a cytotoxic activity (depending on HR deficiency) and antitumor immune activity, which might be more relevant on HR proficient cells^38–41^. This hypothesis should be further investigated in the HR proficient population to provide NLR as a marker able to identify those patients who can rely on their reactive antitumor-immune response to benefit from PARPi.

We also found that high NLR in patients with primary advanced OC is predictive of larger tumor burden (expressed as LPS-PIV score) and those with higher NLR have higher chances of receiving NACT instead of PDS, compared with those with lower NLR. These observations are in line with the negative prognostic value of high NLR reported in other retrospective series of different OC settings^30,42^. Finally, our data confirm that NLR at baseline has an independent prognostic impact for both PFS and OS.

Our cohort's strength relies on collecting data from a large single-center population consecutively enrolled in a prospective study for tissue-BRCA status investigation. Furthermore, no data about NLR according to BRCA status in OC have been published before. However, it should be recognized that neutrophils and lymphocytes counts are non-specific parameters because they could be influenced by concurrent conditions, such as infections or inflammation. The cut-off value to discriminate between the high or low group using NLR is not clearly established. We decided to use the cut-off closer to the median value, which was 4 in our population, as it has been previously proposed^43^ and considering that the more often used values are 3 or 5^19,44^. In particular, in a previous meta-analysis of 12 studies including patients with ovarian cancer, an elevated preoperative NLR was associated with more advanced stage and worse OS and PFS^30^; interestingly, all the included studies used different neutrophil-to-lymphocyte ratio cut-offs (ranging from 2.1 to 4), and finally, the Authors concluded that a neutrophil-to-lymphocyte ratio ≥ 3 is associated with poorer survival.

Of course, the lack of a definitive cut-off might be a critical limitation in our assessment's general application, though it would reasonably not change the final findings of our analysis.

In conclusion, NLR is confirmed to be a prognostic marker in OC patients. The information obtained from our study has revealed a potential new biologic subtype of BRCA patients, correlated with inflammation status and easily detectable. Next research should be focused on the role of NLR with regard to PARPi and ICIs response, regardless of BRCA/HRD status, underlining others less common but not less effective mechanisms of action of these drugs and allowing further personalization of treatment.

## Materials and methods

### Patients

Between January 2017 and December 2019, newly diagnosed high grade serous ovarian cancer (HGSOC) patients with FIGO Stage IIIC-IV, admitted at the Gynecologic Oncology Unit, Fondazione Policlinico A. Gemelli IRCCS in Rome were consecutively tested for the tissue/blood BRCA mutation within a prospective study^45^.

All women received gynecologic oncologist counseling before BRCA testing and a signed written informed consent. BRCA-mutations were classified according to the ENIGMA BRCA1/2 Gene Variant Classification Criteria (http://www.enigmaconsortium.org/) and women with variants of uncertain significance (VUS) were considered wild-type. Tissue samples for somatic testing were obtained during surgery. Patients were included if they had a primary diagnosis of high-grade serous ovarian cancer (HGSOC), if they had received 3 weekly carboplatin-paclitaxel as first line treatment, with or without maintenance therapy, if their BRCA mutational status was available. Their blood parameters should have been collected in the local laboratory at Fondazione Policlinico A. Gemelli IRCCS 48 h before staging laparoscopy/laparotomy.

All women gave written informed consent for their data to be collected and analyzed for scientific purposes. The Institutional Review Board of the Catholic University of the Sacred Hear approved the study (CICOG-01-07-19/35).

### Clinical data and follow-up

According to our Institutional model, patients were initially submitted to clinical evaluation, CT-scan and staging laparoscopy (S-LPS)^46^ to be triaged to primary debulking surgery (PDS) or neoadjuvant chemotherapy (NACT). Intraperitoneal tumor burden was evaluated at diagnosis using a laparoscopic predictive-index value (LPS-PIV)^46^, classifying women as having: low tumor load in the presence of LPS-PIV < 8, and high tumor load when an LPS-PIV ≥ 8 was observed. A maximal surgical effort was attempted in all patients selected for PDS, and the residual tumor was recorded. The Complexity of surgical procedures in patients receiving PDS was graded according to the surgical complexity score (SCS) by Aletti et al.^47^. Regardless of upfront treatment strategy, all women received six cycles of carboplatin (area under the curve [AUC] 5 or 6) plus paclitaxel (175 mg/mq) every 21 days (Q3W); maintenance therapy was also administrated as indicated according to internal protocol and included Bevacizumab (15 mg/kg) in combination with chemotherapy and maintenance Q3W for 22 cycles or Olaparib 300 mg tablets orally two times per day until disease progression or toxicity for a maximum of 2 years, in BRCA mutated patients (since April 2019).

After treatment administration, patients were entered into a routine follow-up program including gynecological examination, CA125 assessment and CT-scan every 6 months.

Data from medical records were consecutively collected including medical history, surgery results, treatment approach, and genetic counseling.

### Endpoints and statistical analysis

NLR was defined as the absolute neutrophils count divided by the absolute lymphocytes count. Neutrophils and lymphocytes count collected within 48 h before staging laparoscopy or laparotomy were taken into consideration. A cut-off value of 4 was adopted to discriminate patients with low (NLR < 4) (Group 1) versus high (NLR > 4) (Group 2) as primary analysis, according to previously published data and median value in the present series^27,48–52^.

The primary endpoint of the study was to investigate if exists a possible correlation between the BRCA status and NLR values in a large high-grade serous OC population; also, we evaluated the correlation between NLR values and clinical parameters of patients with advanced high-grade serous OC, as well as survival outcomes (progression-free survival and overall survival).

Chi-square or Fisher's exact tests were used for comparison of categorical variables.

Regarding survival analysis, PFS was defined as the time elapsed between the date of diagnosis (staging laparoscopy/laparotomy) and recurrence; patients without evidence of progressive disease at the time of the analysis were censored on the date of their last tumor evaluation. Overall survival (OS) was defined as the time interval between the diagnosis and death of any cause. Patients who were no longer alive at the time of the analysis or had been lost to follow-up were censored on the date of their last follow-up visit. PFS and OS were estimated by the Kaplan–Meier method, and curves were compared by the log-rank or Breslow (Generalized Wilcoxon) tests (at a significance level of 5%), as appropriate. Estimated hazard ratios (HRs) and their two-sided 95% confidence intervals (95% CIs) were calculated using the Cox proportional-hazard model. All statistical calculations were carried out using SPSS 26.0 for Mac (SPSS Inc., Chicago, IL, USA).

### Ethical approval

All procedures performed in studies involving human participants were under the ethical standards of the institutional and/or national research committee and with the 1964 Helsinki declaration and its later amendments or comparable ethical standards.
